# The MRI evaluation of nitric-oxide mediated systemic endothelial function and coronary endothelial function in healthy subjects and patients with coronary artery disease

**DOI:** 10.1186/1532-429X-17-S1-O52

**Published:** 2015-02-03

**Authors:** Micaela Iantorno, Sahar Soleimanifard, Angela M Steinberg, Michael Schär, Matthias Stuber, Gary Gerstenblith, Robert Weiss, Allison Hays

**Affiliations:** 1 Cardiology, Johns Hopkins University, Reston, VA, USA; 2 Critical Care, National Institute of Health, Bethesda, MD, USA; 3 Centre Hospitalier Universitaire Vaudois, Lausanne, Switzerland

## Background

Endothelial cell release of nitric oxide (NO) is one indicator of vascular health. Coronary arteries develop atherosclerotic disease, while left internal mammary arteries (IMA), which are frequently used as bypass conduits in patients with disease, do not. Endothelial cell function of vessels can be assessed by the response to isometric handgrip exercise (IHE); normal function is evidenced by an increase in cross sectional area(CSA), flow velocity(FV) and blood flow(BF), and an abnormal response by no increase in these variables. Recently, the combination of coronary MRI and isometric handgrip exercise (IHE) was shown to noninvasively quantify coronary endothelial function (CEF). We tested the hypotheses that: 1) IMA vasoreactivity to IHE is measurable using coronary 3T MRI 2) endothelial function of IMA differs from that of coronary arteries in patients with coronary atherosclerotic disease (CAD) whereas in healthy subjects endothelial function of the two vascular beds is similar and 3) the IMA response to IHE is primarily mediated by NO, thus reflecting endothelial function.

## Methods

**Figure 1:**
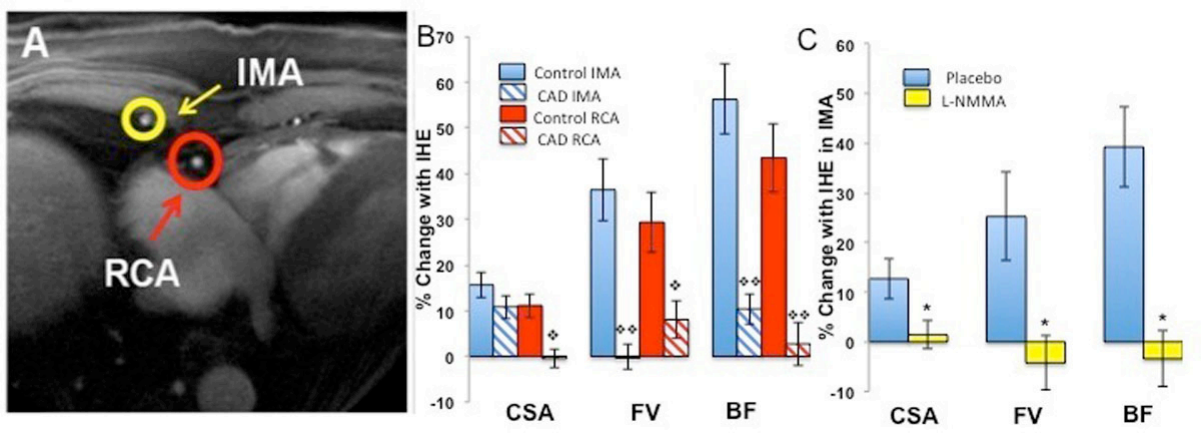
**Panel A:** Examle of a cross section of a right coronary artery (RCA, red circle) and right internal mammary artery (IMA, yellow circle) in same axial imaging plane. **Panel B**. Bar graph representing the % change from baseline in vasoreactive parameters: area, velocity and blood flow with isometric handgrip exercise (IHE) in the IMA and RCA of health controls (N=27, blue) and patients with coronary artery disease (CAD, N=21, red). **Panel C.** L-NMMA (monomethy-L-arginine, NOS inhibitor) study in 9 healthy adults: Summary results for % change from baseline in IMA area, velocity and blood flow changes with IHE for control (blue bars, before L-NMMA) and during L-NMMA infusion (yellow bars), showing that L-NMMA blocks the normal increase in all three vasoreacive measures with stress. Error bars indicate standard error of the mean. ^◊^p<0.001 vs control; ^◊◊^p<0/0001 vs control; *p<0.01 vs baseline; **p<0.0001 vs baseline. CSA: cross-sectional area; FV: flow velocity; BF: blood flow.

We studied 21 CAD patients (60±2years) and 27 healthy subjects (46±4years) on a commercial 3T MRI scanner (Achieva, Phillips, Best, NL) using a 32-element cardiac coil. Anatomical and velocity-encoded spiral cine sequences were obtained of both the right coronary artery (RCA) and an IMA in axial cross-section in the same cine sequence (Fig 1A) at rest and during IHE at 30% of maximum grip strength. Changes from rest to IHE in CSA, FV and BF(BF=CSAxFVx0.5) were quantified. In 9 healthy subjects, the role of NO in the IMA response to IHE was assessed during infusion of the NO synthase inhibitor, monomethyl-L-arginine(L-NMMA,0.3mg/kg/min).

## Results

During IHE in healthy subjects, mean CSA, FV and BF for both RCA and IMA increased significantly from baseline (Fig 1B). As expected, in patients with CAD there was no significant RCA change in CSA, FV or BF with IHE. In the same CAD patients, in contrast, the IMA significantly dilated with IHE (p<0.001) and BF increased (p=0.01, Fig 1B). In contrast to the RCA responses, the CSA changes in the IMA in the healthy individuals and CAD patients did not differ, although the IMA FV and BF responses did (Fig 1B). L-NMMA infusion significantly attenuated the IHE response of the IMA for CSA, FV and BF, indicating a critical role of endothelial-derived NO (Fig 1C).

## Conclusions

Both coronary and systemic endothelial function can now be measured in a single MRI acquisition. The IMA response to IHE is significantly attenuated by L-NMMA indicating the IMA vasoreactive response to IHE primarily reflects NO-dependent endothelial function. Coronary and systemic vascular beds display heterogeneous responses, which promise unique pathophysiologic insights in atherosclerotic and atherosclerosis-free vascular beds.

## Funding

NIH/NHLBI grants R01HL084186, AHA SDG 5200004, AHA 12PRE11510006.

